# Plant-Inspired Layer-by-Layer Self-Assembly of Super-Hydrophobic Coating for Oil Spill Cleanup

**DOI:** 10.3390/polym11122047

**Published:** 2019-12-10

**Authors:** Liping Ding, Yanqing Wang, Jinxin Xiong, Huiying Lu, Mingjian Zeng, Peng Zhu, Haiyan Ma

**Affiliations:** 1 School of Chemistry and Chemical Engineering, Nantong University, Nantong 226007, China; dingliping@ntu.edu.cn (L.D.); a1835948675@163.com (J.X.); luhuiying722@163.com (H.L.); z1743552875@163.com (M.Z.); 2 Department of Materials Science & Engineering, National University of Singapore, Singapore 117575, Singapore

**Keywords:** plant-inspired chemistry, self-assembly strategy, surface modification, super-hydrophobicity

## Abstract

A versatile, facile, energy-saving, low-cost and plant-inspired self-assembly strategy was used to prepare super-hydrophobic coating in this study. Concretely, an appealing super-hydrophobicity surface was obtained by designing a molecular building block phytic acid (PA)-Fe (III) complex to anchor the substrate and hydrophobic thiol groups (HT). The facile and green modification method can be applied to variety of substrates. The as-prepared PA-Fe (III)–HT coated melamine composite sponge possesses both super-hydrophobic and superlipophilicity property. Moreover, it displays superior efficiency to separate the oil–water mixture and splendid oil spill cleanup.

## 1. Introduction

**Figure 1:**
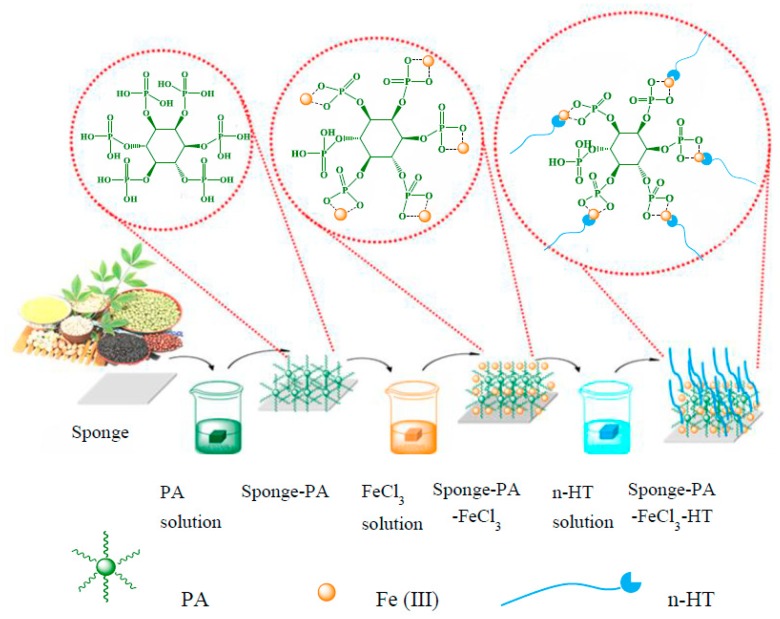
Schematic description of fabricating super-hydrophobic coating on sponges.

The petroleum industry is the basic industry of the national economy. The machinery, electricity, petrochemical, automobile, architecture, and other traditional industries, and even the increasingly important electronic industry and the development of many high-tech industries, all rely on oil resources [1,2]. Oil plays an irreplaceable role in economic development, but it also brings serious environmental pollution problems due to accidents and improper reprocessing in the actual production process [3,4]. The direct combustion method is usually adopted to remove the oil pollutants in the follow-up treatment of the offshore oil spill [5,6]. However, direct burning of oil will not only cause a waste of resources but also pollute the air. If the leaked oil can be separated from seawater and recycled, the problems of resource waste and environmental pollution can be solved well. Based on the insoluble nature of oil and water, the researchers have designed many hydrophobic materials for oil–water separation and recovery in recent years [7,8,9]. Particle materials [10,11,12] such as TiO_2_ and carbon spheres, two-dimensional materials such as planar membranes, nanofibers, and hollow fibers [13,14,15,16], and three-dimensional materials [17,18,19,20] such as sponges and foam have been used in oil–water separation experiments. The particle materials with a large surface area can quickly absorb a thin layer oil phase on the water surface, but their low absorption capacity and difficult collection limit their application in the oil–water separation field. After the surface modification of the two-dimensional materials, the hydrophobicity of the film surface can be obtained, and the oil–water mixture can be separated continuously under the action of gravity. Unfortunately, the two-dimensional film materials must be in a specific device in order to play its role and is not suitable for the treatment of large-scale oil leakage at sea. Herein, nowadays, oil–water separation materials are mainly focused on three-dimensional porous hydrophobic materials [21,22,23]. Three-dimensional porous hydrophobic materials have a large amount of storage space, which can directly absorb the leaked oil on the sea surface, and its bulk structure is easy to transport after the completion of oil absorption process. On the premise of satisfying the good oil absorption function, ‘fast, simple and easy to release the absorbed oil from the material’ becomes the criterion for the selection of suitable three-dimensional materials [24]. Polymer-based three-dimensional porous materials (such as sponge, foam, and aerogel) are considered to be ideal materials for oil leakage treatment because of their multi-layer network structure, absorption capacity, long penetration channel, and adjustable pore structure. In particular, the polymer has excellent flexibility, which makes it have high elasticity. The absorption oil is discharged by compression, which simplifies the separation process [25]. For a more efficient, cheaper, and readily scalable remediation solution, modified commercially available polymer sponges/foams have recently arisen as a suitable supporting body for oil spills’ remediation [26]. Melamine sponge not only has the characteristics of network interworking structure, high porosity, large specific surface area and low density, but also has good thermal stability and compressibility [27,28,29,30]. After hydrophobic modification, the sponge can not only absorb the oil phase but also release the absorbed oil phase by simple extrusion, which greatly reduces the recovery cost of the oil phase. According to the theory of Wenzel and the Cassie–Baxter model, the hydrophobic properties of the material surface are determined by the chemical properties and the roughness of the material surface [31,32]. Silicon/fluorine materials are common low surface energy material, which has excellent properties such as weather resistance, refractory, corrosion resistance, and oxidation stability [18,33,34,35]. However, silicon/fluorine modified coatings have the problems of high price and a complex preparation process, which limits its large-scale application. Therefore, it is highly desirable to develop a simple and economical method to fabricate melamine sponge with super-hydrophobic qualities for large-scale offshore oil spills. With the scarcity of oil resources, there is a deterioration of the environment and the aggravation of a greenhouse effect. The manufacturing method of the green economy is particularly important. Getting effective ingredients from common animals and plants is a new inspiration for solving these problems. In recent years, inspired by the extraction of raw materials from animals and plants, more and more studies on dopamine [36], levodopa [16], tannic acid [37], gallic acid [38], and lignin [39] have been reported. In this paper, a versatile, facile, energy-saving, low-cost but efficient plant-inspired strategy was developed to prepare the super-hydrophobic melamine sponge (Figure 1). This strategy employs a metal-organic coordination-enabled layer-by-layer (LbL) self-sssembly method to prepare super-hydrophobic coating. Metal-organic coordination structures’ materials contain reticular metal centers and organic linkers. The above two constituents can bind with each other via metal–ligand coordination interaction [40]. Metal-organic coordination endows not only a peculiar combination of hardness and extensibility, but also a covalent-like stability [41]. It would be highly desirable that these properties are introduced into the LbL self-assembly process to acquire the super-hydrophobic coating by a facile and controllable way [42]. Our strategy offers several distinct advantages, including the following: (i) the super-hydrophobic modification surface can be fabricated at room temperature without any harsh preparation condition. (ii) Our method is conducted by using the chelate reaction between PA and Fe (III) and is suitable for a wide variety of substrates. (iii) Plant-derived PA, commercial FeCl_3_, and hexadecyl thiol (HT) are low-cost, so this fabrication technology shows good industrial application potential.

## 2. Experimental

### 2.1. Materials

Melamine sponge, polyurethane sponge, and non-woven fabric were provided from Hart New Materials Research Institute Co., Ltd. (Wuxi, China). Hexadecyl thiol (>99%), FeCl_3_ 6H_2_O, methyl blue, oil red, phytic acid (50 wt % in water), sodium chloride, sodium hydroxide, ethanol (99.5%), toluene (>99.8%), petroleum ether (>99%), dichloroethane (>99%), and hydrochloric acid (32%) were purchased from Sigma-Aldrich (Shanghai, China). Gasoline, diesel oil, heat-conductive silicone oil and corn oil were purchased from a local store. All chemicals were used as received without further purification.

### 2.2. Layer by Layer Self-Assembly Super-Hydrophobic Modification of Various Highly Hydrophilic Polymer Materials

The pristine melamine sponge (size of 3 cm × 3 cm × 2 cm, porosity > 99%), polyurethane sponge (size of 3 cm × 3 cm × 2 cm, porosity > 95%) and non-woven fabric (size of 3 cm × 2 cm) were washed sequentially with distilled water and ethanol under ultrasonic conditions for about 30 min and then dried in an oven at 60 °C. The dry substrates were immersed in the PA solution (5 g/L, 50 mL) for 2 min first, and then the excess solution was squeezed to remove unabsorbed PA. After that, the substrates were immersed into 50 mL of a 0.1 mol/L of FeCl_3_·6H_2_O solution for another 1 min and then the excess solution was squeezed to remove the excess FeCl_3_·6H_2_O solution. So far, a self-assembly layer has been completed. Similarly, the multiple-cycle assembly was achieved by repeating cycles of alternately dipping the substrates into the PA aqueous solution and the FeCl_3_·6H_2_O solution. There are no washing steps between different cycles. In this experiment, for melamine sponge modification, a maximum of 10 cyclic experiments have been done. After completing the multiple-cycle assembly, the PA-FeCl_3_ coated substrates were rinsed with deionized water and dried at 60 °C. The PA-FeCl_3_-HT coated substrates were achieved by immersing the dried substrates into an ethanol solution containing 1 wt% HT for 24 h, then rinsed with ethanol and deionized water in turn and finally dried at 80 °C for 4 h.

### 2.3. Characterizations and Measurements

#### 2.3.1. Structure and Morphology Characterizations

Infrared spectra of the melamine sponge, polyurethane sponge and non-woven fabric were performed by Fourier Transform Infrared spectroscopy (FT-IR) (Bruker, Karlsruhe, Germany) under the attenuated total reflection (ATR) mode. Surface morphologies of the pristine and modified melamine sponge, polyurethane sponge and non-woven fabric were characterized by scanning electron microscopy (SEM, ZEISS, Evo-10, Jena, Germany). The element mapping texts of the modified melamine sponge were characterized by scanning electron microscopy (SEM, ZEISS, Sigma-300, Jena, Germany) and energy-dispersive spectroscopy (EDX). The contact angle tests of the pristine and modified polymer materials were measured by a contact angle measuring system (Dataphysics, OCA20, Stuttgart, Germany).

#### 2.3.2. Oil/Water Separation Experiments

The prepared hydrophobic melamine sponge was immersed in various oil phases or organic solvents and then quickly taken out. The oil absorption capacity was determined according to the mass change of the material before and after absorption. It was calculated using Equation (1):

(1) $$\mathrm{AC}=\frac{W_{a}-W_{b}}{W_{b}}$$

*W*_b_ and *W*_a_ are the weight of melamine sponge before and after oil absorption, respectively. The recycling ability of the hydrophobic material is reflected according to the repeated absorption extrusion test. Firstly, the material is saturated and absorbed, and then the absorbed oil phase is released by external force extrusion. The weight of each absorption extrusion cycle was taken to determine the recycling absorption capacity of the material.

## 3. Results and Discussion

**Figure 2:**
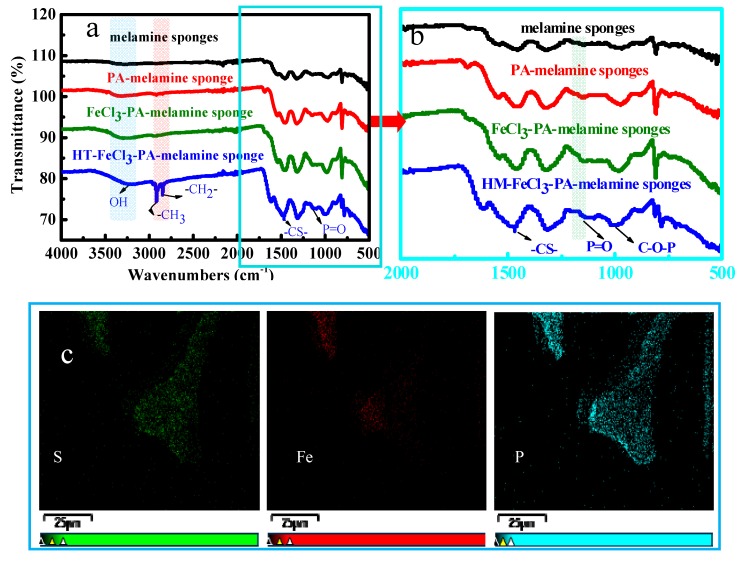

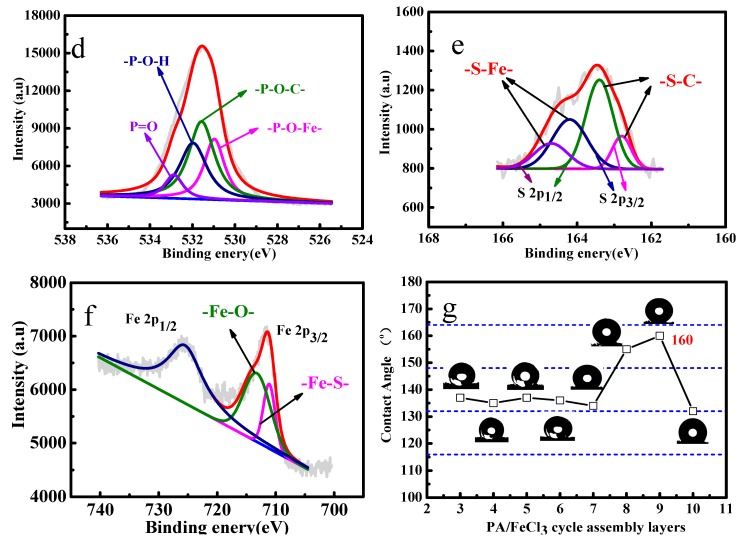
The FT-IR spectra (**a**,**b**), EDX map (**c**), O1s (**d**), S2p (**e**), and Fe2p (**f**) are level spectra of the PA-Fe (III)–HT-sponge, the contact angle of multiple-cycle assembly of modified melamine sponge (**g**).

Figure 2 describes the structural changes of modified melamine sponge by layer-by-layer self-assembly strategy. Figure 2a,b are the FT-IR spectra of modified melamine sponge. Figure 2b is a local magnification graph of Figure 2a. After being coated with PA layer, two characteristic peaks of PA at 1145 cm^−1^ (P = O) and 3328 cm^−1^ (–OH) are observed (Figure 2a red curve). The absorption intensity of P = O groups becomes significantly weak after being cross-linked with Fe (III) ion, and –OH peak at 3328 cm^−1^ becomes a little stronger (Figure 2a,b green curve). After hydrophobic modification, the characteristic absorption peaks of HT at 1471 cm^−1^ (–CS–), 2848 cm^−1^ (–CH_2_–) and 2925 cm^−1^ (–CH_3_) appear (Figure 2a,b blue curve) [43]. These results confirm the successful incorporation of the hydrophobic function HT on the surface of the sponge. EDX mapping images (Figure S1: The initial image before mapping) of the PA-Fe (III)–HT–sponge further reveal the existence and uniform distribution of S, P, and Fe elements on the sponge surface (Figure 2c). Notably, these FT-IR and EDX figures only demonstrate the introduction of PA-Fe (III)–HT, and X-ray photoelectron spectroscopy (XPS) measurement was employed to provide more information about the interactions between the PA-Fe (III)–HT layers. The survey scan spectra and the chemical composition of the modified melamine sponge are presented in Figure S2. The existence of the absorption peaks at 133.6 eV (3.36%) (P2p), 165.5 eV (1.48%) (S2p), and 711.2 eV (2.42%) (Fe2p) of the PA-Fe (III)–HT–sponge demonstrates that the phosphate groups and long-chain alkyl thiol were successfully introduced into the modified layer and further cross-linked with Fe (III) ion. The O, S, and Fe core-level spectra of modified melamine sponge are shown in Figure 2d–f. As shown in Figure 2d, the O1s spectrum of the modified melamine sponges could be divided into P = O (532.8 eV), –P–O–H (531.9 eV), –P–O–C– (531.5 eV), and –P–O–Fe– (530.9 eV). In Figure 2e, the S2p core-level spectrum (167.6 eV and 163.7 eV) represents –S–Fe and –S-C absorption peaks, respectively. The Fe2p core-level spectrum (Figure 2f) is divided into two characteristic functional groups –Fe–O and –Fe–S, respectively. To ensure the complete coverage of the original substrate surface and obtain a well-designed surface morphology with suitable micro/nano hierarchical roughness, the sponge was dipped into modification solutions several times. Figure 2g is the contact angle of multiple-cycle assembly of modified melamine sponge (repeat cycles of alternately dipping the substrates into the PA aqueous solution and the FeCl_3_·6H_2_O solution), the hydrophobicity of the modified sponge with nine cycles has the best hydrophobicity. The reason may be that HT needs to compete with PA to coordinate with ferric iron. Therefore, the super-hydrophobic property of the coating can be controlled by changing the concentration of iron ions (layer number of assembly) participating in the reaction. Based on the proper multiple-cycle assembly of PA-Fe (III) coating (nine cycles), the sponge was further modified by thiol-terminated, showing super-hydrophobicity.

**Figure 3:**
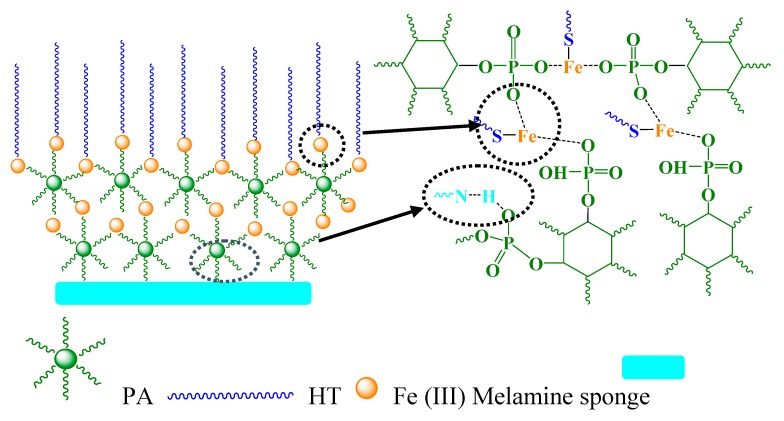
Chemical binding mechanism in the layer-by-layer assembly process.

The layer-by-layer assembly process can be further explained by Figure 3. PA contains six phosphonic acid groups that can be used as an organic ligand to bind well versatile substrates, including quartz, silicon, glass, Al, stainless steel, polycarbonate, etc. [44]. In this paper, when the melamine sponge was first immersed in PA solution, the PA molecules could be absorbed and immobilized on the sponge substrate by covalent and/or non-covalent bonding by utilizing hydroxyl groups from phosphate group and the –NH_2_ group of the sponge surface. As the sponge was subsequently immersed into the Fe (III) solution, the PA-Fe (III) complexes aggregations would be further formed on the substrate to provide the appropriate surface topography and roughness for constructing super-hydrophobic surfaces (Figure S3). As is well-known, the wetting behavior of a surface is mainly related to both its surface topography and chemical composition. Therefore, the sponge was further modified with thiol-terminated molecules through the metal-thiol coordination, grafting the hydrophobic long-chain onto the PA-Fe (III) coating surface to achieve the hydrophobic modification of the substrate.

**Figure 4:**
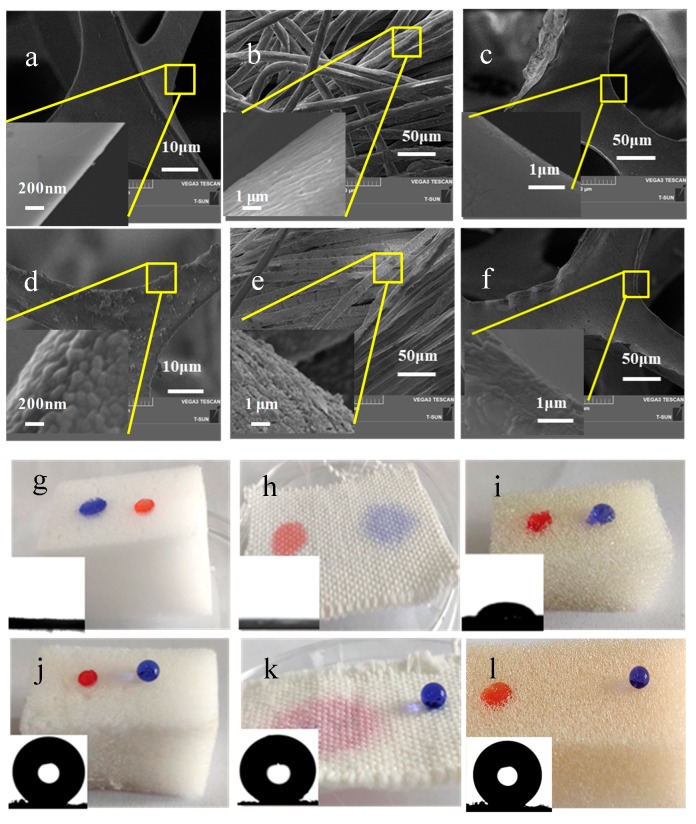
SEM images (**a**–**f**) and digital photos (**g**–**l**) of melamine sponge (**a**,**d**,**g**,**j**), polyurethane sponge (**b**,**e**,**h**,**k**) and non-woven fabric (**c**,**f**,**i**,**l**) before and after PA-Fe (III)–HT treatment.

Figure 4 shows the evolutive images in the surface morphology of modified substrate by the scanning electron microscopy (SEM) at different magnifications. For the pristine melamine sponge without PA-Fe (III)–HT treatment, it is clearly seen that the sponge is smooth and flat (Figure 4a). There is no micro/nano rough structure on the surface. However, for the melamine sponge after PA-Fe (III)–HT treatment, the SEM image displays that the sponge surface possesses randomly rough structures at the micro-nanometer scale (Figure 4d). Not only melamine sponge, but also the non-woven fabric (Figure 4b,e) and polyurethane sponge (Figure 4c,f) were also fabricated successfully using this facile method, and their morphologies also present similar changes. SEM results demonstrate that the PA-Fe (III)–HT treatment results in the formation of micro/nanoscale hierarchical structures on the sponge surface, and it is very important for the final super-hydrophobicity. Figure 4g–l are comparative pictures of hydrophilicity substrate before and after PA-Fe (III)–HT treatment. As shown in the figure, the pristine superhydrophilic melamine sponge (Figure 4g), non-woven fabric (Figure 4h) and hydrophilic polyurethane sponge (Figure 4i) become super-hydrophobic (Figure 4j–l) after being coated with PA-Fe (III) aggregations, followed by HT modification. Water droplets retain a spherical shape on the surfaces of the treated porous materials and all the water contact angles (WCAs) of samples are more than 150°.

**Figure 5:**
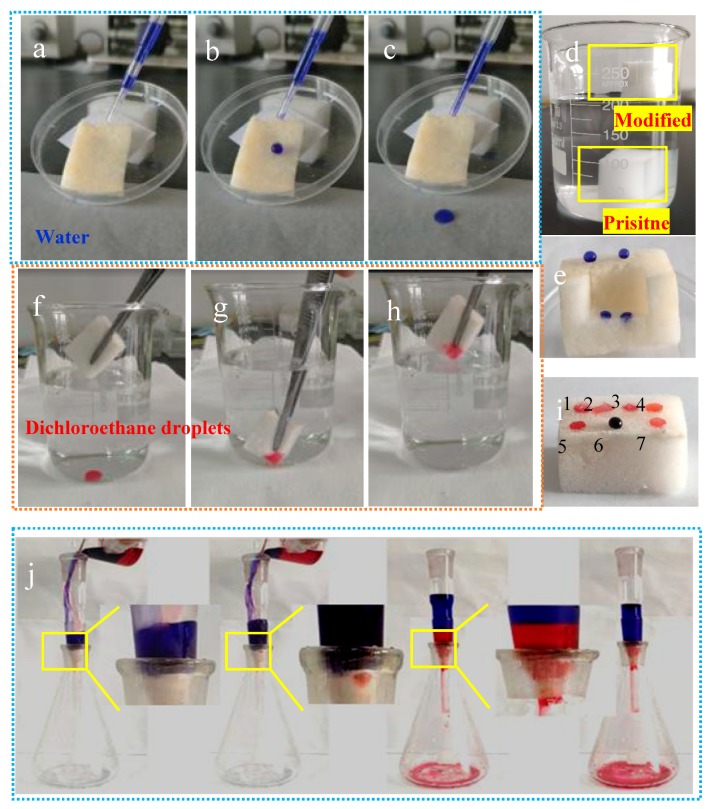
Rolling photos of water droplets on modified melamine sponges (**a**–**c**), the state of melamine sponges in water (**d**), wettability of modified and unmodified melamine sponge (**e**), dichloroethane droplets absorption (**f**–**h**), absorption of different kinds of oil/organic solvent (1. toluene, 2. silicone oil, 3. gasoline, 4. diesel oil, 5. corn oil, 6. water, and 7. petroleum ether) by modified melamine sponge (**i**) and separation of an oil/water mixture via modified melamine sponges (**j**).

To further investigate the hydrophobic properties and oil absorption ability of the coatings, a series of experiments were carried out. As shown in Figure 5a–c, the water droplets can be quickly rolled from the surface of the modified melamine sponge without leaving any marks (see Movie S1). Figure 5d shows that the pristine melamine sponge sinks to the bottom of the beaker while the modified melamine sponge floats on the water surface. Figure 5e indicates that it can be clearly seen that water cannot infiltrate the surface of the modified melamine sponge; on the contrary, water can completely infiltrate the internal unmodified melamine sponge. The reason is that the modified melamine sponge has the micro-nano-scale structure and the hydrophobic chemical property, and the as-prepared sample surface repels the water drops. As shown in Figure 5f–h, when the modified melamine sponge makes contact with the oil droplets that are submerged in water, it completely absorbs the droplet in an instant (see Movie S2). In addition, the modified melamine sponge has absorption properties for different kinds of oil besides dichloromethane (Figure 5i). Figure 5i also intuitively shows the lipophilic and hydrophobic characteristics of the modified sponge. Moreover, the modified melamine sponge could efficiently separate dichloromethane/water mixture solely under gravity (Figure 5j, see Movie S3).

**Figure 6:**
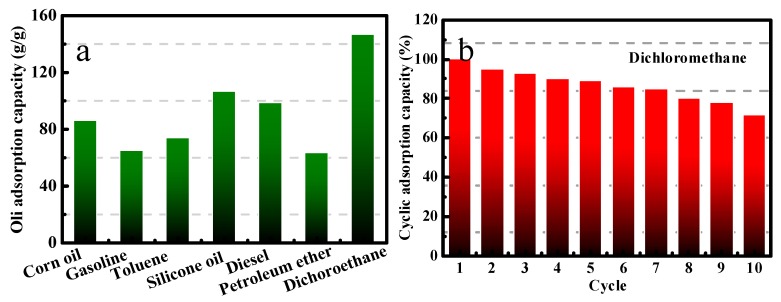

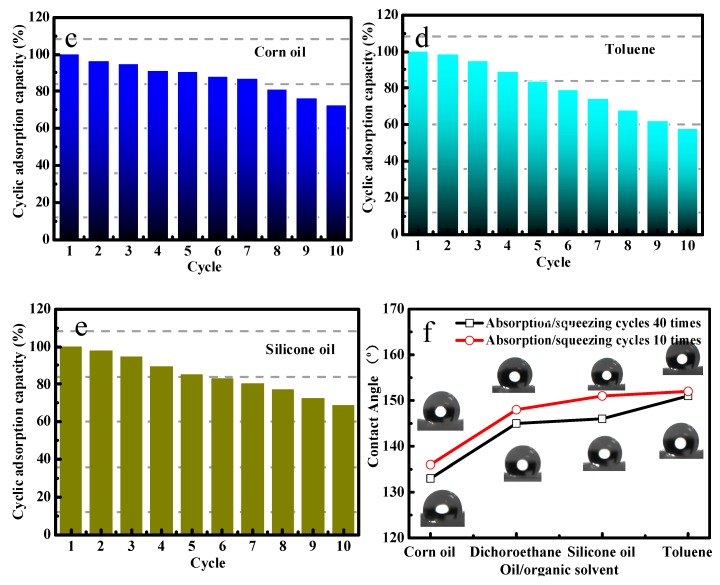
The absorption capacity (**a**) and cyclic serviceability (**b**–**e**) of the super-hydrophobic melamine sponge for various types of oils and organic solvents; (**f**) the water contact angle of the modified sponge after absorbing and extruding different oil/organic solvent for 10 and 40 cycles.

Figure 6 is a qualitative measurement of the absorption capacity of a modified melamine sponge for oil/organic solvent. As shown in Figure 6a, the modified sponge exhibits excellent absorption capacity towards a wide range of organic solvents and oils, even in the case of high-density solvents (such as dichloromethane) and viscous oils (such as corn oil and silicone oil) can be easily recycled by gently squeezing. The modified sponges can absorb 60–150 times its dry weight in the above solvents and oils. The weight ratio of absorbed solvent/sponges not only depends on the density and viscosity of the oils/organic solvents, but also may be related to the swelling of polymers in some solvents. The excellent oil/water separation ability is further confirmed by the reusability of modified sponges for different oil/organic solvent. The melamine sponge will be subjected to repeated stress extrusion and porous structure collapse; thus, absorption capacity will be reduced. Figure S4 shows the destruction of the melamine sponge skeleton after multiple extrusion. However, the modified sponges still keep a strong absorption capacity after 10 absorption–desorption cycled times (Figure 6b–e). Figure 6f shows the water contact angle of the modified sponge after absorbing and extruding different oils/organic solvent for 10 and 40 cycles. As shown in Figure 6f, the water contact angle of the modified sponge changes little after repeatedly absorbing and extruding different oils/organic solvent, and still displays the super-hydrophobic characteristics. These results indicate that the facile construction of modified sponge with special wettability has a broad application prospect in multi-purpose cycle absorption of oil/organic solvent and has great practical application potential. The excellent oil absorption and recycling property of modified sponges result from the chemical modification of surface hydrophobicity and the construction of micro-nanoscale hierarchical structures on the sponge surface, which is very important for the final super-hydrophobicity, just like the surface of the lotus leaf (Figure S5). Combining roughness on both the micro- and nanoscale results in a hierarchically structured surface, which is the basis of the Lotus effect. The lowest area of contact between the droplet and the surface occurs in the case of the hierarchical structure, which can be expected to have super-hydrophobicity. In addition, the multiscale roughness allows for more stable air pocket formation, guarding against destabilizing factors on both the micro and nanoscale.

## 4. Conclusions

In conclusion, a versatile, facile, green, and low-cost surface assembly strategy was designed based on plant-inspired chemistry to prepare super-hydrophobic surfaces. The micro/nano hierarchical roughness structure was first obtained by fabricating micro-nano phytic acid metal complexes’ aggregations, and then the super-hydrophobicity surface will be achieved by grafting thiol-containing hydrophobic groups on the above rough surface by a metal–thiol coordination effect. This facile method can be employed to prepare various super-hydrophobic materials. Furthermore, taking the PA-Fe (III)–HT coated melamine sponges as an example, the super-hydrophobic melamine sponges can be successfully used to collect oil spills and display excellent separation efficiency.

## Supplementary Materials

The following are available online at https://www.mdpi.com/2073-4360/11/12/2047/s1, Figure S1: The initial image before mapping, Figure S2: The XPS wide-scan spectrum of modified melamine sponge, Figure S3: The SEM image of the PA-Fe complex deposit, Figure S4: SEM images: a. unsqueezed melamine sponge, b. squeezed melamine sponge (10 cycles) and c. squeezed melamine sponge (40 cycles), Figure S5: Schematic of the hydrophobic model inspired by the surface of lotus leaves, Movie S1: The water droplets rolled from the surface of the modified melamine sponge, Movie S2: The dichloroethane droplets absorption of the modified melamine sponge, Movie S3: The separation of an oil/water mixture via modified melamine sponges.
