# Supplementary material for: Plant-Inspired Layer-by-Layer Self-Assembly of Super-Hydrophobic Coating for Oil Spill Cleanup
Source: Polymers (Basel). 2019 Dec 10;11(12):2047. doi: 10.3390/polym11122047 (PMC6960575; doi:10.3390/polym11122047)
Supplement: Supplementary file 1 [file polymers-11-02047-s001.pdf]

Plant-inspired Layer-by-Layer Self-assembly of Super-hydrophobic Coating for Oil Spill Cleanup

Liping Ding[1#], Yanqing Wang[1,2*#], Jinxin Xiong[1], Huiying Lu[1], Mingjian Zeng[1], Peng Zhu[1*] and Haiyan Ma[1*]

1. School of Chemistry and Chemical Engineering, Nantong University, Nantong 226007, P. R. China
2. Department of Materials Science & Engineering, National University of Singapore, 117575, Singapore

# Liping Ding and Yanqing Wang contributed equally to this work

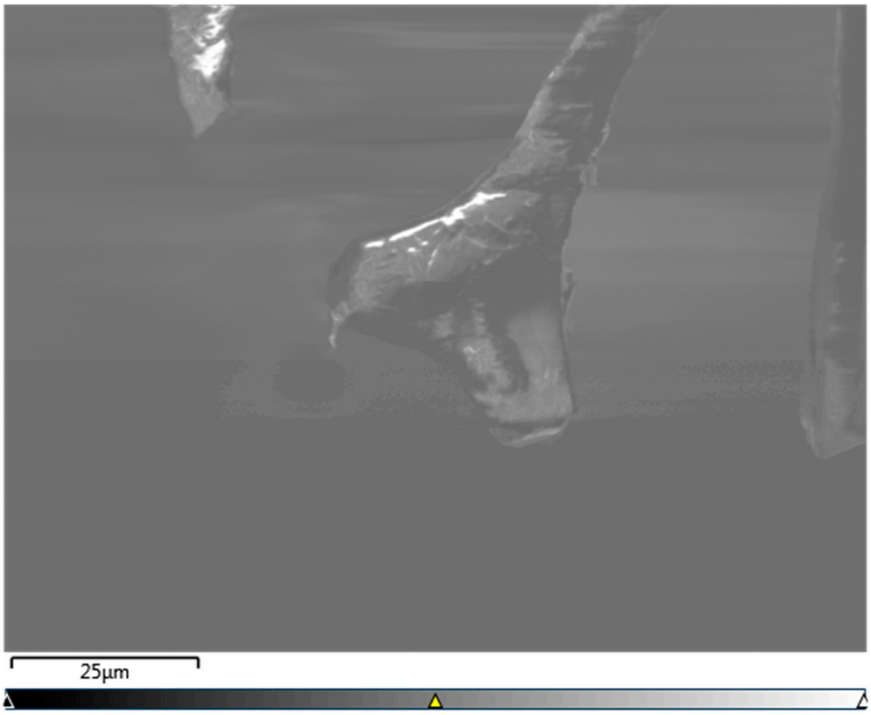

Fig.S1 The initial image before mapping

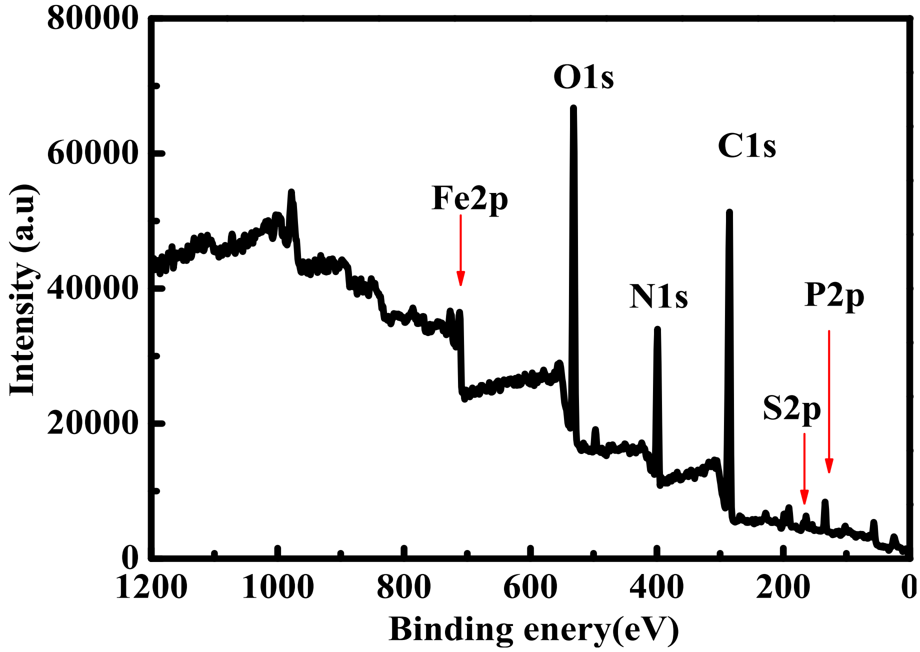

Fig.S2 The XPS wide-scan spectrum of modified melamine sponge.

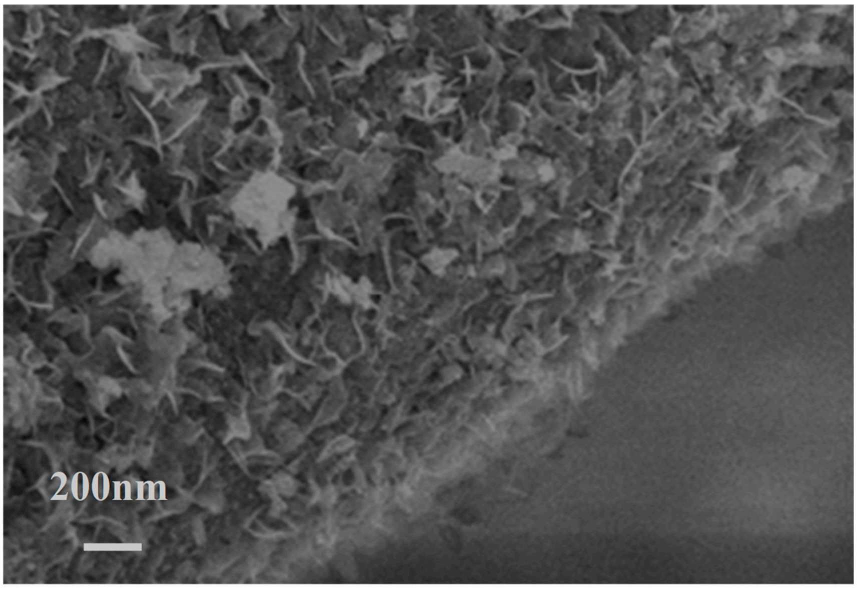

Fig. S3 The SEM image of the PA-Fe complex deposit.

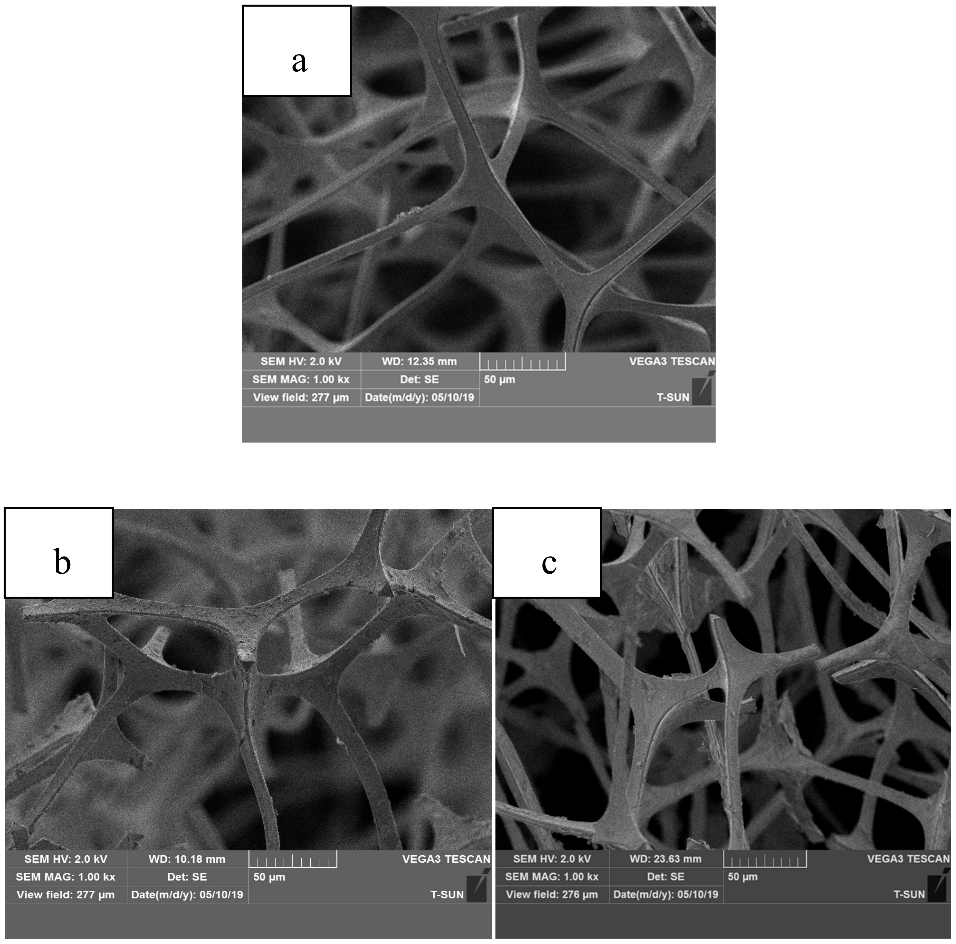

Fig.S4 SEM images: a. unsqueezed melamine sponge, b. squeezed melamine sponge (10 cycles) and c. squeezed melamine sponge (40 cycles).

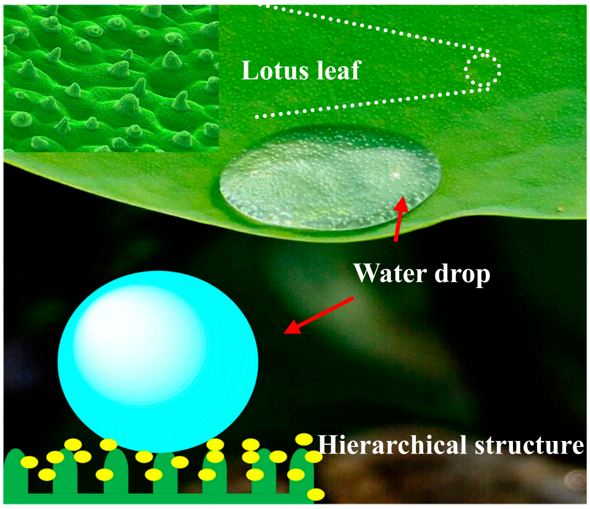

Fig.S5 Schematic of the hydrophobic model inspired by the surface of lotus leaves

.
